# Supplementary material for: Genomic deletion of Bcl6 differentially affects conventional dendritic cell subsets and compromises Tfh/Tfr/Th17 cell responses
Source: Nat Commun. 2024 Apr 30;15:3554. doi: 10.1038/s41467-024-46966-6 (PMC11061177; doi:10.1038/s41467-024-46966-6)
Supplement: Supplementary file 3 — Description of Additional Supplementary Files [file 41467_2024_46966_MOESM3_ESM.pdf]

**Description of Additional Supplementary Files**

File Name: Supplementary Data 1
Description: Expression levels of top 50 DEGs between control cDC1 and control cDC2

File Name: Supplementary Data 2
Description: DEGs between control and *XCR.Bcl6*$^{KO}$ cDC1 and control and *CD11c.Bcl6*$^{KO}$ cDC2

File Name: Supplementary Data 3
Description: Expression levels of top 50 DEGs between ESAM$^{hi}$ and ESAM$^{lo}$ cDC2

File Name: Supplementary Data 4
Description: Reagent list
