# Supplementary material for: Genomic deletion of Bcl6 differentially affects conventional dendritic cell subsets and compromises Tfh/Tfr/Th17 cell responses
Source: Nat Commun. 2024 Apr 30;15:3554. doi: 10.1038/s41467-024-46966-6 (PMC11061177; doi:10.1038/s41467-024-46966-6)
Supplement: Supplementary file 8 — Reporting Summary [file 41467_2024_46966_MOESM8_ESM.pdf]

nature portfolio

Corresponding author(s): Katharina Lahl
Dirk Baumjohann

Last updated by author(s): Feb 25, 2024

Reporting Summary

Nature Portfolio wishes to improve the reproducibility of the work that we publish. This form provides structure for consistency and transparency in reporting. For further information on Nature Portfolio policies, see our Editorial Policies and the Editorial Policy Checklist.

Statistics

For all statistical analyses, confirm that the following items are present in the figure legend, table legend, main text, or Methods section.

n/a	Confirmed	
[ ]	[x]	The exact sample size (*n*) for each experimental group/condition, given as a discrete number and unit of measurement
[ ]	[x]	A statement on whether measurements were taken from distinct samples or whether the same sample was measured repeatedly
[ ]	[x]	The statistical test(s) used AND whether they are one- or two-sided<br>*Only common tests should be described solely by name; describe more complex techniques in the Methods section.*
[x]	[ ]	A description of all covariates tested
[ ]	[x]	A description of any assumptions or corrections, such as tests of normality and adjustment for multiple comparisons
[ ]	[x]	A full description of the statistical parameters including central tendency (e.g. means) or other basic estimates (e.g. regression coefficient) AND variation (e.g. standard deviation) or associated estimates of uncertainty (e.g. confidence intervals)
[ ]	[x]	For null hypothesis testing, the test statistic (e.g. *F*, *t*, *r*) with confidence intervals, effect sizes, degrees of freedom and *P* value noted<br>*Give P values as exact values whenever suitable.*
[x]	[ ]	For Bayesian analysis, information on the choice of priors and Markov chain Monte Carlo settings
[x]	[ ]	For hierarchical and complex designs, identification of the appropriate level for tests and full reporting of outcomes
[x]	[ ]	Estimates of effect sizes (e.g. Cohen's *d*, Pearson's *r*), indicating how they were calculated

Our web collection on statistics for biologists contains articles on many of the points above.

Software and code

Policy information about availability of computer code

Data collection	FACS Melody, FACSAria III, FACSAria Fusion, Fortessa X20, LSRFortessa or Symphony flow cytometer (all BD Biosciences), Zeiss LSM710 and LSM900 confocal microscope (Zeiss), TriStar plate reader, Pannoramic MIDI II scanner (3DHISTECH), Cellcounter XP-300 (Sysmex).
Data analysis	STAR aligner (v2.7.6a), Gene Transfer Format (Ensembl GTF GRCm38.101), DESeq2 (v1.30.1), Ensembl's BiomaRt (v2.46.3), pheatmap (v1.0.12), R environment (v4.0.5), Venny (v2.1), PlotMA, PCAtools (v2.2.0), ggpubr (v0.4.0), clusterPro ler (v3.18.1), GeneRatio, ZEN software (Zeiss), 'marls Viewer (Oxford Instruments), CaseViewer software (v2.4), Graphpad Prism (v9.4.0, GraphPad) (v9.0), FACSDiva software (BD Biosciences)' FlowJo software (v.10.8.1, TreeStar)

For manuscripts utilizing custom algorithms or software that are central to the research but not yet described in published literature, software must be made available to editors and reviewers. We strongly encourage code deposition in a community repository (e.g. GitHub). See the Nature Portfolio guidelines for submitting code & software for further information.

Data

Policy information about availability of data

All manuscripts must include a data availability statement. This statement should provide the following information, where applicable:

- Accession codes, unique identifiers, or web links for publicly available datasets
- A description of any restrictions on data availability
- For clinical datasets or third party data, please ensure that the statement adheres to our policy

Raw RNA-sequencing data files on splenic XCR1 and CD11c.Bcl6KO mice are accessible through the NCBI SRA database under the accession number PRJNA834905.
ESAMhi and ESAMlo gene sets derived from GSE76132 (Lau, C. M. et al. Leukemia-associated activating mutation of Flt3 expands dendritic cells and alters T cell responses. Journal of Experimental Medicine 213, 415–431 (2016))
Flow cytometry raw data available upon request.

Research involving human participants, their data, or biological material

Policy information about studies with human participants or human data. See also policy information about sex, gender (identity/presentation), and sexual orientation and race, ethnicity and racism.

Reporting on sex and gender	N/A
Reporting on race, ethnicity, or other socially relevant groupings	N/A
Population characteristics	N/A
Recruitment	N/A
Ethics oversight	N/A

Note that full information on the approval of the study protocol must also be provided in the manuscript.

Field-specific reporting

Please select the one below that is the best fit for your research. If you are not sure, read the appropriate sections before making your selection.

- [x] Life sciences
- [ ] Behavioural & social sciences
- [ ] Ecological, evolutionary & environmental sciences

For a reference copy of the document with all sections, see nature.com/documents/nr-reporting-summary-flat.pdf

Life sciences study design

All studies must disclose on these points even when the disclosure is negative.

Sample size	Sample sizes were not predetermined. We performed every experiment at least two times with at least 3 mice each. We describe the exact numbers of animals/Samples for each experiment in the figure legends.
Data exclusions	One out of five spleens per genotype did not yield good cuts and was therefore excluded from confocal analysis. For the antibody ELISAs and the day 14 NP-KLH immunization experiment, one mouse per genotype was removed due to the complete absence of a response, indicating that the immunization failed. For the in vivo cross presentation, one wt mLN sample was removed due to abnormally large lymph nodes. For the in vitro cross-presentation assays, one CD11c tg spleen sample, one wt mLN sample, and two CD11c tg mLN samples were removed due to known pipetting errors. In Fig. 9B, one wt and one tg were removed due to a suspected switch in genotypes. In Citrobacter infection experiments for the day 9 readout, one wt and one tg mouse had to be removed due to a weight loss exceeding 20% at day 3 post injection. For supplementary data reported using the Clec9a data, 5 control mock, 2 tg mock, 1 control infected and 2 tg infected were removed due to unspecific tomato reporter expression. For Citrobacter experiments with a d21 readout, one wt mouse had to be removed due to a weight loss exceeding 20% at day 3 post injection.
Replication	Numbers of replications for each experiments are stated in the Figure legends.
Randomization	During the study, we randomly chose the mice from the same littermates for each experiment group. We also randomly chose the age- and sex- matched littermates for control groups.
Blinding	Genotypes were blinded for histology assessment. Other readouts were not performed in a blinded manner, as those readouts were not subjective. Gating of FACS data was consistent within each experiment.

Reporting for specific materials, systems and methods

We require information from authors about some types of materials, experimental systems and methods used in many studies. Here, indicate whether each material, system or method listed is relevant to your study. If you are not sure if a list item applies to your research, read the appropriate section before selecting a response.

Materials & experimental systems

n/a	Involved in the study
☐	☒ Antibodies
☐	☒ Eukaryotic cell lines
☒	☐ Palaeontology and archaeology
☐	☒ Animals and other organisms
☒	☐ Clinical data
☒	☐ Dual use research of concern
☒	☐ Plants

Methods

n/a	Involved in the study
☒	☐ ChIP-seq
☐	☒ Flow cytometry
☒	☐ MRI-based neuroimaging

Antibodies

Antibodies used	All antibodies used, including catalogue number and dilution, are added as a suppl. table to the manuscript.
Validation	All the antibodies used were validated according to the manufacturers instructions. We tested all antibodies prior to the study starting with the recommended dilution and including fluorescent minus one controls.

Eukaryotic cell lines

Policy information about cell lines and Sex and Gender in Research

Cell line source(s)	We used heat killed OVA-expressing mouse embryonic fibroblasts, which were gifted to us by Caetano Reis e Sousa. The cells and method were destribed previously, and we did not perform any further testing or characterization of the cells (Sancho, D. et al. Identification of a dendritic cell receptor that couples sensing of necrosis to immunity. Nature 458, 899–903 (2009)).
Authentication	We did not independently authenticate the cell line.
Mycoplasma contamination	The cell line was not tested for mycobacteria in house.
Commonly misidentified lines (See ICLAC register)	N/A

Animals and other research organisms

Policy information about studies involving animals; ARRIVE guidelines recommended for reporting animal research, and Sex and Gender in Research

Laboratory animals	Xcr1.cre (B6-Xcr1tm2Ciphe), CD11c.cre31 and Clec9a.cre mice were crossed to Bcl6fl/fl mice (from Alexander Dent or JAX.org stock number #023727) to obtain DC-specific Bcl6-knockout models. Clec9a.Bcl6KO mice and Clec9a.Bcl6CONTROL control mice were both maintained homozygous for cre and additionally contained homozygous a Rosa26fl-Stop-fl-YFP allele (JAX.org stock number 006148). CD11c.Bcl6 and XCR1.Bcl6 models were maintained heterozygous for cre and negative littermates were used as control controls. Additional strains used in this study were B6.SJL-Ptprca Pepcb/BoyJ (congenic C57BL/6.CD45.1 mice from the Jackson Laboratory), C57BL/6-Tg (TcraTcrb)1100Mjb/J and B6.Cg-Tg(TcraTcrb)425Cbn/J (OT-I and OT-II mice from Jackson Laboratory). Mice were group-housed in individually ventilated cages, maintained on a 12 h light and dark cycle at 22 °C and 55% humidity, and maintained under specific pathogen-free conditions. Euthanization was performed using cervical dislocation without anesthesia. Experimental groups were sex and age-matched. Male and female littermates were used between 8 and 15 weeks of age, RNA-Seq was performed on female mice only.
Wild animals	N/A
Reporting on sex	Both male and female litter-mates were used and the genotypes are depicted by the use of different symbols throughout the manuscript. RNA-Seq was performed on female mice only.
Field-collected samples	N/A
Ethics oversight	All animal experiments were performed in accordance with European regulation and federal law of Denmark and Germany. Permission was granted either approved by the Danish Animal Experiments Inspectorate, the Regierung von Oberbayern or the Landesamt für Natur-, Umwelt und Verbraucherschutz NRW.

Note that full information on the approval of the study protocol must also be provided in the manuscript.

Plants

Seed stocks	N/A
Novel plant genotypes	N/A
Authentication	N/A

Flow Cytometry

Plots

Confirm that:

- [x] The axis labels state the marker and fluorochrome used (e.g. CD4-FITC).
- [x] The axis scales are clearly visible. Include numbers along axes only for bottom left plot of group (a 'group' is an analysis of identical markers).
- [x] All plots are contour plots with outliers or pseudocolor plots.
- [x] A numerical value for number of cells or percentage (with statistics) is provided.

Methodology

Sample preparation	Single cell suspension from specific tissues were prepared as stated in the methods under "cell preperation" and "flow cytometry". Cells were blocked with Fc block (purified anti-CD16/32) and stained for specific markers. For intracellular stainings, cells were live/dead stained using either LIVE/DEAD.'" Fixable Near-IR Dead Cell Stain Kit (Invitrogen), Fixable Viability Dye eFluorTM-780 (Invitrogen) or DRAQ7 (BioLegend) prior to fixation and permeabilization before intracellular transcription factor and/or cytokine staining using the Foxp3 Fixation/Permeabilization kit (eBioscience).
Instrument	FACSAria III, FACSAria Fusion, FACS Melody, Fortessa X20, LSRFortessa or Symphony flow cytometer (all BD Biosciences)
Software	FACSDiva software (BD Biosciences) and Flowio software (v.10.8.1, TreeStar)
Cell population abundance	For cell sorting of splenic DCs, we sorted 2-4x10*4 per sample
Gating strategy	Gating strategies are included in the manuscript as supplementary figures. We started with FSC-A against SSC-A and excluded doublets using FSC-A against FSC-W. Wherever used, boundaries between negative and positive cells were clear due to distinct populations and validated using fluorescent minus one controls.

- [x] Tick this box to confirm that a figure exemplifying the gating strategy is provided in the Supplementary Information.
